# Leveraging Existing Biodiversity and Zoonosis Monitoring Infrastructure for Integrative Plant Pathogen Surveillance in Natural Ecosystems

**DOI:** 10.3390/insects17040383

**Published:** 2026-04-02

**Authors:** Valeria Trivellone, Andrew J. Mackay, Christopher M. Stone, Christopher H. Dietrich

**Affiliations:** Illinois Natural History Survey, Prairie Research Institute, University of Illinois, Champaign, IL 61820, USA; amackay@illinois.edu (A.J.M.); cstone@illinois.edu (C.M.S.); chdietri@illinois.edu (C.H.D.)

**Keywords:** biodiversity, integrated wildlife surveillance, leafhoppers, One Health, plant pathogen

## Abstract

Outbreaks of emerging and re-emerging diseases in both animals and plants are increasing due to climate change, globalization, land-use change, and agricultural intensification. While most pathogen monitoring programs focus on zoonotic threats, wildlife and other organisms in natural habitats can also serve as reservoirs and sentinels for pathogens of agricultural and ecological concern. Plant communities and the pathogens circulating within them are underrepresented in integrated disease monitoring frameworks. This study demonstrates how biodiversity and zoonosis monitoring programs conducted in protected habitats (tallgrass prairies and woodlands) across Illinois, together with insect specimens preserved in biorepositories, can be leveraged to improve knowledge of the identities and ecological associations of a wide range of potential pathogens. We developed an integrative workflow combining taxonomic identification, molecular screening, and epidemiological inference to detect vector-borne plant pathogens from archived insect material. Focusing on Hemiptera (Auchenorrhyncha), we screened specimens for phytoplasmas (Mollicutes), uncultured bacterial plant pathogens transmitted by sap-feeding insects, and characterized host–pathogen associations. At least three distinct phytoplasma strains were detected: ‘*Candidatus* Phytoplasma asteris’ (16SrI-B), ‘*Candidatus* Phytoplasma pruni’-related strains (16SrIII), and ‘*Candidatus* Phytoplasma sacchari’-related strains (16SrXI-H). The latter represents the first documented occurrence of a 16SrXI-H phytoplasma subgroup in Illinois. Overall, we identified five insect specimens harboring phytoplasmas across four preserved sites, all of which were previously unreported for insect–phytoplasma associations. These findings demonstrate the value of existing biodiversity and zoonosis monitoring infrastructures for proactive surveillance of plant pathogens and extending the One Health paradigm to explicitly include natural ecosystems.

## Simple Summary

Disease outbreaks affecting plants and animals are becoming more frequent as a result of climate change, global trade, and changes in land use. Most disease monitoring programs focus on threats to humans and livestock, while far less attention is given to pathogens circulating in natural ecosystems or plant pathogens. In this study, we show how insects collected in protected natural areas, together with specimens stored in biological collections, can be used to detect plant pathogens that circulate in natural areas and track them before they cause outbreaks. Leveraging biodiversity and zoonosis monitoring programs, we screened sap-feeding insects collected from prairies and woodlands in Illinois for the detection of cryptic plant-infecting bacteria. We detected three different plant pathogenic bacteria, including one that had never before been reported in Illinois, and identified five new insect–pathogen associations. Rather than predicting disease outbreaks directly, our goal is to improve basic knowledge of where pathogens occur and how they interact with insects and plants in natural habitats. This information is essential for proactive disease prevention and strengthens a One Health approach that links environmental, agricultural, and ecosystem health.

## 1. Introduction

**Figure 1:**
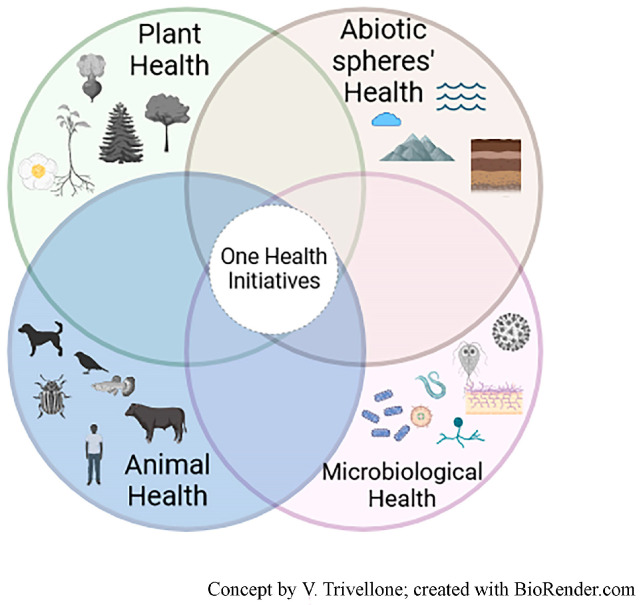
Tetrahedron representation of a balanced vision of One Health consisting of four equivalent components. The concept of ‘microbiological health’ is introduced referring to the stability and functioning of microbial communities within their environmental context, where microbes are known to be of crucial importance interacting with other biological components (e.g., gut microbiomes in animals and endophytes in plants) and with abiotic components (e.g., cycling nutrients, enhancing soil structure, and altering atmospheric oxygen levels). This figure was created with BioRender.com.

One Health (OH) is a simple yet powerful framework for addressing emerging infectious diseases, as it seeks to build consensus around integrated solutions that safeguard health across interconnected biological systems [1]. However, OH has also been criticized as conceptually and operationally incomplete, often remaining anchored to targeted response-based approaches rather than more holistic and proactive system understanding [2]. In 2022, the One Health High-Level Expert Panel proposed a working definition intended to integrate principles from related frameworks such as EcoHealth and Planetary Health. This definition emphasizes the interdependence of human, animal, plant, and environmental health, and calls for coordinated action across sectors, disciplines, and societal levels to promote sustainable development and resilience [3]. Despite its integrative ambition, the operationalization of this definition remains challenging. In particular, its broad wording risks obscuring an epistemic gap between how the biosphere is perceived and managed and what is required to understand its underlying biological functioning. This gap limits critical awareness of systemic drivers of health and disease and fosters the perception of health crises as unpredictable or accidental. Addressing this gap requires critical learning processes, including the re-evaluation of inherited paradigms, context-specific knowledge generation, and the development of operational frameworks that counteract phenomena such as the ‘epistemology of ignorance’ [4]. A further tension facing One Health frameworks is the need to maintain and promote a range of discipline-specific expertise (e.g., taxonomic), while fostering and building capacity for interdisciplinary collaborations in collection and analysis of data. The commonly adopted representation of One Health as a triad linking humans, animals, and the environment further reflects anthropocentric framing, in which the environment is often reduced to a passive backdrop rather than an active, living system. Building on hybrid perspectives proposed by Furberg [5], we adopt a biosphere-centered view in which humans are embedded within, rather than positioned above, interconnected plant, microbial, animal, and abiotic components (Figure 1). From this perspective, health outcomes emerge from shared ecological space and interdependence, implying that management and surveillance strategies must operate in balance with the functioning of the entire biosphere.

Emerging infectious diseases (EIDs) are increasingly recognized as predictable yet highly complex phenomena, arising from land-use change, agricultural intensification, biodiversity loss, and climate-driven environmental shifts. Rather than rare or stochastic events, the emergence of disease is now widely understood as the outcome of altered ecological opportunities that favor pathogen transmission and host switching. From this perspective, the One Health (OH) framework is particularly relevant for complex epidemiological systems such as vector-borne pathogens, the transmission dynamics of which are inherently shaped by interactions among hosts, vectors, and the environment. It has been repeatedly documented that many pathogens circulate silently within alternative or secondary hosts, frequently associated with natural or semi-natural ecosystems, where they remain largely undetected [6,7,8]. These enzootic or cryptic transmission cycles often persist without notice until pathogens spillover into humans or into species on which humans depend, such as crops or livestock. Within a One Health perspective, effective surveillance must, therefore, acknowledge the biosphere as an interconnected system and recognize that EIDs more often arise from changes in ecological opportunity rather than true pathogen novelty [9]. Recent studies have highlighted the value of integrated approaches to wildlife disease monitoring. For example, Barroso et al. [10] demonstrated how integrated wildlife monitoring in Europe can identify disease maintenance hotspots and key drivers of interspecific transmission. Similar conceptual and operational needs were emphasized by Cardoso et al. [11] and Machalaba et al. [12], who argued for closer integration between biodiversity science and health surveillance. Despite these advances, biodiversity monitoring and medical surveillance still rarely interact, even though they target the same biological space and often the same organisms.

The disconnection between disease surveillance and biodiversity monitoring is particularly evident for plant diseases. As with many animal (including human) pathogens, plant pathogens are most frequently detected in managed landscapes, such as agricultural fields and urban or peri-urban environments, where their impacts become economically or socially visible. By the time such pathogens are detected, opportunities for outbreak prevention are often limited. In this sense, managed ecosystems function as epidemiological observatories, revealing pathogen dynamics that have long remained undocumented in natural systems. In contrast, natural ecosystems are still poorly characterized from a plant pathogen perspective. Plant pathologists typically sample natural areas opportunistically, if at all, resulting in major knowledge gaps regarding pathogen diversity, host range, and circulation in wild plant and vector communities. This lack of baseline information severely constrains spillover risk assessment and predictive capacity, particularly under ongoing environmental change.

In this context, we aim to address this epistemic gap by proposing a joint surveillance framework that integrates biodiversity monitoring with pathogen detection. By leveraging existing biodiversity inventories, biorepositories, and monitoring programs for zoonotic disease surveillance at the Prairie Research Institute (University of Illinois at Urbana-Champaign), we outline an operational workflow for detecting, characterizing, and contextualizing pathogens circulating in wildlife. Using phytoplasmas (non-yet-culturable bacteria within the class Mollicutes) and their insect hosts as a biological model, we integrate host–pathogen research with natural history collections to evaluate the potential spillover risk and strengthen One Health surveillance in natural and semi-natural ecosystems. Our objective is not to predict emergence events per se, but to improve baseline knowledge of a pathogen’s presence and ecological context as a prerequisite for proactive and preventive One Health implementation.

## 2. Materials and Methods

### 2.1. Leveraged Monitoring Programs and Specimen Collection

**Figure 2:**
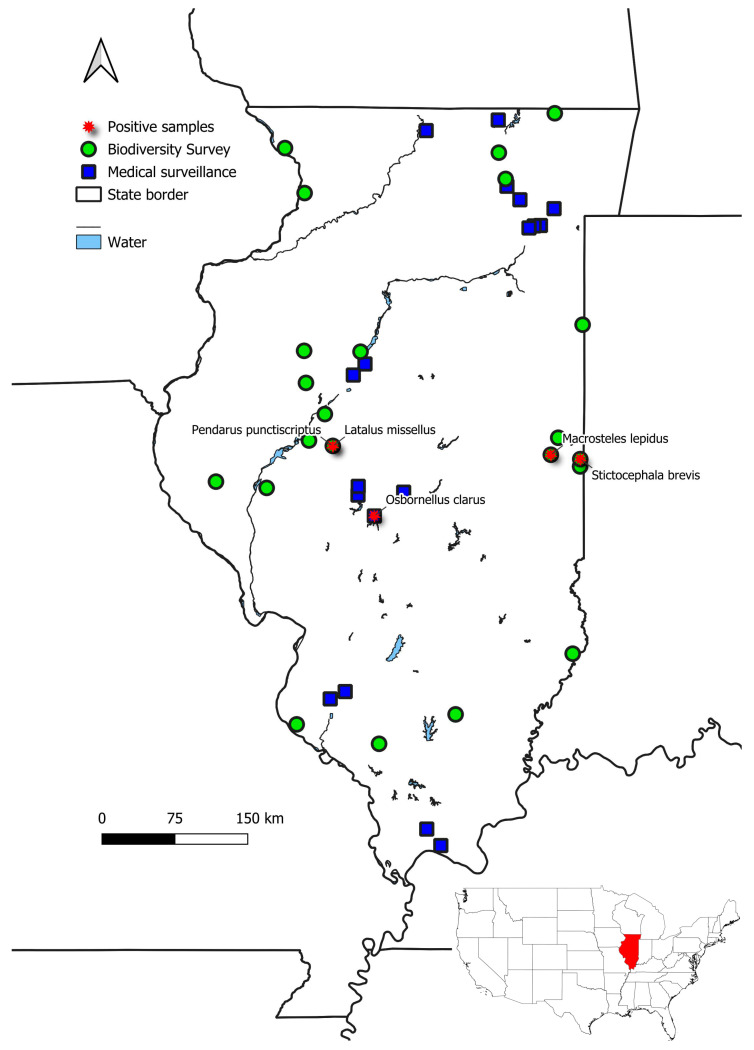
Distribution of the sites surveyed during the biodiversity surveys in 2021 and 2025 (green circle) and the public health (medical) surveillance surveys in 2022 and 2024 (blue quadrat) in Illinois, USA. The stars in red represent positive specimens, and the names of the species are plotted. The map was created using QGIS 3.10; the inset at the bottom right shows the location of Illinois within the contiguous United States. Major rivers and lakes are shown in light blue.

Independent monitoring programs are conducted annually at the Prairie Research Institute (University of Illinois at Urbana-Champaign, USA) to document both arthropod diversity and pathogen presence in natural and managed ecosystems across Illinois. In this study, we leveraged specimens collected through two distinct, but complementary monitoring efforts led by the Illinois Natural History Survey (INHS, Prairie Research Institute): (i) biodiversity surveys conducted by the INHS Leafhopper Lab, and (ii) public health surveillance conducted by the INHS Medical Entomology Lab. Biodiversity surveys focus on sampling Auchenorrhynchan insects (Hemiptera) across natural habitats, primarily tallgrass prairies, with the main objective of documenting the decline in prairie specialists and endangered species. The samples screened in this study were obtained through surveys conducted in 2021 and 2025, for a total of 22 sites across the state (Figure 2, represented by green circles). Specimens are collected using various standard entomological methods, including sweeping and vacuuming of vegetation, nighttime collection at lights, and in Malaise (flight intercept) traps.

Zoonotic disease surveillance monitoring primarily targets mosquito populations in urban parks, forest preserves, and other natural or semi-natural areas representing a gradient of residential land use and green space. This program aims to investigate vectors of zoonotic pathogens of public health and veterinary concern in Illinois. Mosquitoes and other hematophagous Diptera are collected using multiple trap types baited with dry ice, including CDC light traps (model 512), miniature ultraviolet (UV, model 1012) light traps (John Hock Company, FL, USA), and BG Sentinel-2 traps (Clarke Mosquito Control Products, Inc., St. Charles, IL, USA) supplemented with additional attractant cues [UV-LED light source, BG-Lure^®^ (Clarke Mosquito Control Products, Inc.) and/or 1-Octen-3-ol (Woodstream Corporation, Inc., Lancaster, PA, USA)]. Traps are operated for approximately 24 h per sampling event, after which specimens are transported on dry ice and stored at −80 °C until processing (see Mackay et al. [13] for full methodological details). In addition to mosquitoes, these traps incidentally collect other insect orders and arthropods. The samples screened in this study were collected from woodland habitats in 2022 and 2024, for a total of 18 sites across the state (Figure 2, blue quadrats). Future efforts may expand this to screen a larger number of specimens collected during mosquito sampling from 2023 to 2025 at 31 additional natural areas, encompassing a diverse range of habitats across Illinois.

All Auchenorrhynchan specimens, including putative vectors of phytoplasmas (obligate vector-borne plant pathogens), were repurposed for phytoplasma screening and entered a specific workflow (see below and Supplementary Table S1).

### 2.2. Non-Destructive DNA Extraction

Immediately after sampling, all specimens were preserved in 96% ethanol and stored in −20 °C freezers at INHS facilities until nucleic acid extraction. A maximum of 4 individuals per species were tested for the presence of phytoplasmas. DNA was extracted using a non-destructive lysis protocol allowing molecular screening while retaining voucher specimens for deposit in permanent collections at INHS [14].

Briefly, individual specimens were incubated at 56 °C for two days in a lysis buffer containing Tris-HCl, EDTA, and SDS, supplemented with Proteinase K (20 mg/mL) to facilitate tissue digestion. DNA purification was performed using an iminodiacetic acid-based chelating resin (20% working solution). After removal of the insect body, an equal volume of chelating resin solution was added to the lysate. A final 30 min incubation at 56 °C completed the extraction. DNA extracts were stored at −20 °C or immediately analyzed for the presence of phytoplasmas.

### 2.3. Voucher Preparation, Host Association and Metadata Integration

Following DNA extraction, specimens were pinned, labeled, and assigned a unique INHS collection code. Vouchers were subsequently housed in standard entomological drawers within archival-quality collection cabinets at the INHS Insect Collection.

Associated metadata, including collection locality, date, collector, taxonomic identification, preservation status, INHS collection code, and infection status, were recorded in the INHS Insect Collection Database [15]. The digitized host–pathogen associations stored in the collection database constitute a documented, publicly accessible scientific record that can be revised as taxonomy is updated or as additional data become available.

### 2.4. Real-Time PCR Screening for Phytoplasmas and Conventional PCR Assays

Phytoplasma detection was performed using a universal probe-based quantitative PCR (qPCR) assay targeting the *16S rRNA* gene. Reactions consisted of a commercial qPCR master mix containing hot-start Taq DNA polymerase, dNTPs, optimized buffer, and stabilizers. Amplification used a universal primer set (p16S-fw: 5′-CGTACGCAAGTATGAAACTTAAAGGA-3′; p16S-rv: 5′-TCTTCGAATTAAACAACATGATCCA-3′) and a hydrolysis probe labeled with a 5′ FAM reporter and dual 3′ quenchers ZEN and IBFQ (5′-FAM-TGACGGGAC-ZEN-TCCGCACAAGCG-IBFQ-3′). Ultrapure nuclease-free water was used for all reaction mixtures. Each qPCR plate included a phytoplasma-positive control to validate assay performance and monitor amplification efficiency. The reaction mixture without DNA templates was included as a negative control.

Amplifications with conventional nested PCR assays were carried out on the 16S–23S ribosomal RNA gene for all qPCR-positive samples, yielding amplicons of about 1240 bp for the subsequent characterization of the phytoplasma strain by sequencing. The universal primer pairs for phytoplasmas P1/P7 [16,17], followed by R16F2n/R2 [18], were used. Tubes with the reaction mixture devoid of DNA templates were included in each experiment as a negative control. As a positive control for both qPCR and PCR, we used DNA of the FD (Italian grapevine yellows) phytoplasma, provided by the Viticulture Research Centre (Conegliano, Italy). The PCR products were analyzed using 1% precast agarose gels with SYBR Safe DNA gel stain electrophoresis.

### 2.5. Sequencing, Phytoplasma Characterization and Phylogenetic Analysis

Amplicons obtained were purified through the PureLink PCR Purification Kit (Invitrogen, Waltham, MA, USA) and sequenced directly by the commercial sequencing facilities of DNA Service, Roy J. Carver Biotechnology Center, University of Illinois at Urbana-Champaign, US. Bidirectional sequencing of each amplicon was carried out using a primer-walking approach, employing the PCR primers and internal primers (P1/P7 and R16F2n/R2) to obtain complete sequence coverage. Raw sequences were trimmed for the unwanted 5′ and 3′ fragments generally characterized by low sequence quality and assembled using Staden v2.0 [19]. All sequences obtained in this study were deposited in NCBI under the accession numbers listed in the ‘Data availability’ paragraph and in Table 1.

Potential chimeric sequences were assessed using the DECIPHER package (v3.4) in R [20]. Assembled Sanger consensus sequences were screened using a reference-guided chimera detection approach (FindChimeras) against phytoplasma reference sequences. No chimeric sequences were detected. Phytoplasma taxonomic assignment was carried out using the *iPhyClassifier* platform (https://plantpathology.ba.ars.usda.gov/cgi-bin/resource/iphyclassifier.cgi, accessed on 16 December 2025), which applies internal classification modules specifically designed for phytoplasma 16Sr group and subgroup determination.

Sequences from the same gene were aligned with MEGA v7 [21], and the MUSCLE algorithm [22] was used for sequence alignments.

A maximum likelihood phylogenetic analysis was conducted using the RaxML v2.0 (Randomized Axelerated Maximum Likelihood) program [23] under the GTR + Γ+I model of nucleotide substitution, with among-site heterogeneity rates modeled using a gamma distribution and the proportion of invariant sites estimated by maximum likelihood. Branch support was assessed using 1000 bootstrap replicates, with bootstrap support values mapped onto branch lengths. Reference strains used in the phylogenetic tree are reported in Supplementary Material Table S2.

## 3. Results

### 3.1. Phytoplasma Screening and Insect Museum Vouchers

Although the INHS biodiversity and zoonotic disease surveillance programs were designed for distinct ecological and public health objectives, and they are not focused directly on plant pathogen surveillance, the interoperability of curated specimens, associated metadata, and molecular workflows enabled a cross-sector surveillance approach consistent with One Health principles.

**Figure 3:**
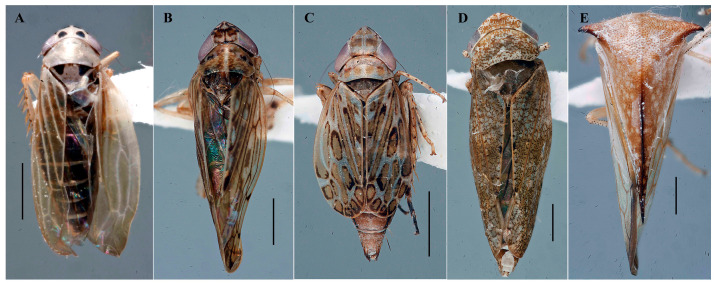
Dorsal views of the 4 species of leafhoppers (**A**–**D**) and 1 treehopper (**E**) that tested positive for phytoplasmas. (**A**) *Macrosteles lepidus* (Van Duzee, 1894) (Illinois Natural History Survey Insect collection code, 1086365); (**B**) *Osbornellus clarus* Beamer, 1937 (1083960); (**C**) *Latalus missellus* (Ball, 1899) (1083966); (**D**) *Pendarus punctiscriptus* (Van Duzee, 1892) (1083969); and (**E**) *Stictocephala brevis* (Walker, 1851) (1089637). Scale bar 1.0 mm.

All specimens screened in this study (646 individuals belonging to 141 species, Supplementary Table S1) were dry-point-mounted following DNA extraction and are preserved as vouchers in the INHS Insect Collection. Despite molecular processing, the bodies of all specimens remained largely intact, allowing confident taxonomic assignment at the species level using traditional morphological criteria based on the inspection of the male genital structures (Figure 3). Notably, specimens recovered as bycatch from mosquito surveillance traps showed fully intact bodies, enabling both molecular screening and morphological identification. The incidental capture of non-target insects, including Hemiptera, in mosquito surveillance traps has been documented previously and examination of such bycatch can yield valuable information that would otherwise go undetected, including new occurrence and distribution data for non-target taxa such as phytoplasmas [24,25,26,27,28,29].

Among the screened material, 16 specimens tested positive for the presence of phytoplasma using qPCR, but only 5 yielded amplifiable DNA for downstream sequencing (Supplementary Table S3). These specimens were identified as *Macrosteles lepidus* (Van Duzee, 1894), *Osbornellus clarus* Beamer, 1937, *Latalus missellus* (Ball, 1899), *Pendarus punctiscriptus* (Van Duzee, 1892), and *Stictocephala brevis* (Walker, 1851). Representative images of each positive specimen are shown in Figure 3. Voucher specimens are deposited under INHS collection codes 1086365, 1083960, 1083966, 1083969, and 1089637 (Table 1).

### 3.2. BLAST Analysis, Phytoplasma Identification, and Phylogenetic Analysis

Five phytoplasma-positive samples yielded amplicons with sizes ranging from 1242 to 1669 bp (Supplementary Material Table S2). *iPhyClassifier* and BLAST v2.13.0 analyses classified the five phytoplasma strains detected in three distinct ribosomal groups: 16SrI, 16SrIII and 16SrXI. Virtual RFLP analysis of the *16S rRNA* gene F2nR2 fragment using *iPhyClassifier* assigned the phytoplasma detected in *O. clarus* to 16Sr group I, subgroup B, producing a restriction pattern identical to the reference profile of ‘*Candidatus* (*Ca*.) Phytoplasma (P.) asteris’ (GenBank accession AP006628; similarity coefficient = 1.00). Consistent with this classification, BLAST analysis of the *16S rRNA* gene sequence revealed the highest similarity to members of the ‘*Ca.* P. asteris’ clade, sharing 99.84% sequence identity to phytoplasma strains previously reported from *Vitis vinifera* in Pennsylvania, USA (GenBank accessions KX236145 and KX236147), and to a phytoplasma detected in the putative vector *Amblysellus necopinus* from Mexico (PV564083).

**Figure 4:**
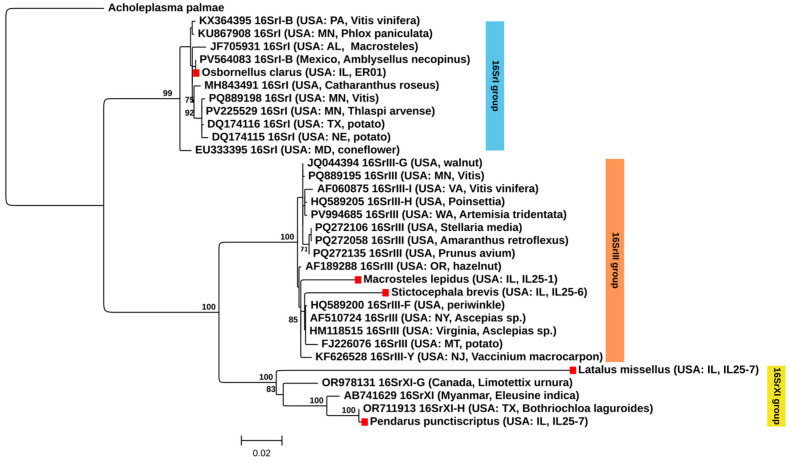
Phylogenetic tree inferred by maximum likelihood (ML) based on *16S rRNA* gene. Bootstrap values > 70% (1000 replicates) are shown. Samples from this study are indicated with a red square. *Acholeplasma palmae* was used as the outgroup (GenBank accession number NR_029152.1).

Phylogenetic analysis based on the 16S–23S rRNA gene region placed the phytoplasma sequence detected in *O. clarus* within the 16SrI clade, clustering with reference strains of ‘*Ca.* P. asteris’ from the eastern and Central United States (Figure 4). The sequence grouped most closely with phytoplasma strains detected in insect hosts from Mexico and the United States: *A. necopinus* and *Macrosteles* sp. (PV564083 and JF705931). This insect-associated clade was separated from lineages comprising phytoplasmas detected in plant hosts, including *Thlaspi arvense*, *Vitis* spp., potato and coneflower, collected in neighboring states, with strong bootstrap support (99%).

Two phytoplasmas belonging to ‘*Ca.* Phytoplasma pruni’-related strains (16SrIII) were independently detected in *M. lepidus* and *S. brevis* at two nearby sites in eastern Illinois. Virtual RFLP analysis using *iPhyClassifier* assigned the phytoplasma detected in *M. lepidus* to the 16SrIII group. The virtual restriction pattern was most similar to the reference profile of subgroup 16SrIII-J (GenBank accession AF147706), with a similarity coefficient of 0.98, indicating that this strain represents a variant of the 16SrIII-J subgroup. Consistent with this classification, BLAST analysis of the *16S rRNA* gene sequence revealed the highest similarity to phytoplasma strains belonging to the 16SrIII clade, supporting its placement within this group. In particular, this strain shared 98.47% with a strain detected in a wild savanna plant, *Aegiphila verticillata*, collected in Brazil (KT148597) assigned to 16SrIII-J subgroup [30], as well as in cultivated *Corylus avellana* collected in Oregon, USA (AF189288), assigned to 16SrIII-B subgroup [31]. A lower similarity of 98.16% was observed with a strain assigned to subgroup 16SrIII-B and detected in *Euscelis incisa* (MN047259), which is a natural vector of 16SrIII-B phytoplasma [32].

In contrast, virtual RFLP analysis of the phytoplasma detected in *S. brevis* produced a restriction pattern that was distinct from all previously established 16Sr group III subgroups. The most similar reference pattern corresponded to subgroup 16SrIII-F (GenBank accession AF510724), but with a similarity coefficient of 0.89, which is below the accepted threshold (≤0.97, [33]) for subgroup assignment. This result suggests that the phytoplasma detected in *S. brevis* may represent a putative new subgroup within the 16SrIII group. BLAST analysis confirmed affiliation with the 16SrIII clade and showed its highest sequence similarity to a strain reported from milkweed collected in New York, USA, assigned to the 16SrIII-F subgroup (HQ589200.1). The strains from *M. lepidus* and *S. brevis* showed 98.31% sequence similarity to each other.

Phylogenetic reconstruction indicated that the *S. brevis* phytoplasma was clustered with reference strains assigned to the 16SrIII-F subgroup, whereas the strain detected in *M. lepidus* formed a sister lineage to this clade (Figure 4).

Two additional phytoplasma strains belonging to ‘*Ca.* Phytoplasma sacchari’-related strains were detected in *L. missellus* and *P. punctiscriptus* collected during the same sampling event at a site in western Illinois. BLAST analysis revealed that the phytoplasma sequence detected in *L. missellus* shared only 95.67% pairwise sequence similarity with a phytoplasma strain previously detected in *Psammottetix cephalotes* from Germany (HQ589192). Due to insufficient sequence coverage, virtual RFLP analysis could not be reliably performed for the phytoplasma detected in *L. missellus*, precluding formal subgroup designation for this strain, even though it was assigned to the ‘*Ca.* Phytoplasma sacchari’-related strain. In contrast, the phytoplasma detected in *P. punctiscriptus* yielded a longer sequence, allowing robust classification. This strain showed the highest sequence similarity (99.78%) to a phytoplasma previously reported from the wild prairie grass *Bothriochloa laguroides* collected in Texas (OR711913, [34]), the reference strain of subgroup 16SrXI-H. Virtual RFLP analysis using *iPhyClassifier* produced a restriction pattern identical to the 16SrXI-H reference profile (similarity coefficient = 1.00), confirming assignment of this strain to subgroup 16SrXI-H. Phylogenetic reconstruction further supported this placement, with *P. punctiscriptus* phytoplasma clustering with reference strains assigned to the 16SrXI-H subgroup, whereas the strain detected in *L. missellus* diverged from this clade (Figure 4).

### 3.3. Novel Insect–Phytoplasma Associations in Natural Areas

*Macrosteles lepidus* is a macropterous Nearctic leafhopper that is widespread and common in the eastern United States [35]. According to previous studies conducted in tallgrass prairies in Illinois, this species completes more than two generations per year, is associated with at least two plant families, and is more frequently linked to ecotonal or disturbed habitats rather than pristine tallgrass prairies. Accordingly, it has been assigned a moderately low coefficient of conservatism [36]. To our knowledge, *M. lepidus* was not previously reported as a phytoplasma-associated host. Its preferred plant hosts are unknown but likely include sedges or grasses that occur in moist woodlands. In this study, *M. lepidus*, collected in restored tallgrass prairie mixed with woody areas in Illinois (Vermillion County), is reported for the first time to be infected with a ‘*Ca.* P. pruni’-related strain. *Stictocephala brevis* is a macropterous Nearctic membracid that appears to be polyphagous, alternating between woody oviposition hosts and herbaceous foodplants, particularly those belonging to Asteraceae and Fabaceae [37]. Similarly, a ‘*Ca.* P. pruni’-related strain was found infecting *S. brevis* collected in a similar habitat in another location in the same area. The leafhopper species *Osbornellus clarus* is reported in Central and North America [38,39], and tested positive for a strain of ‘*Ca.* P. asteris’. *Latalus missellus* and *Pendarus punctiscriptus* are both largely restricted to native grasslands, where they feed on perennial grasses, although their specific host preferences remain unknown. Both species were infected with the novel state record ‘*Ca.* Phytoplasma sacchari’-related strain.

## 4. Discussion

Pathogen vectors have been recognized as key (sentinel) taxa for tracking elusive pathogens in One Health-centered surveillance programs [40]. Pathogen elusiveness stems from the complex trophic networks present in most natural ecosystems, as well as diagnostic challenges (including asymptomatic hosts or low rates of prevalence at which certain pathogens occur), and epistemic gaps. Our findings demonstrate the potential to leverage existing wildlife and biodiversity monitoring infrastructures for proactive surveillance of plant pathogens within natural ecosystems. By linking ecological, taxonomic, and molecular data, our approach extends the One Health paradigm to explicitly include natural ecosystems, advancing early detection and cross-sector surveillance of emerging pathogens at the wildlife–crop interface.

By repurposing specimens and data generated for biodiversity assessment and medical entomology, we expanded plant pathogen screening efforts without additional field sampling, illustrating the value of museum-enabled research infrastructures for integrative pathogen surveillance. Although mosquito surveillance is primarily designed to assess vectors of human health concern, incidental capture of non-target arthropods, including Hemiptera, has been documented previously and shown to provide valuable biodiversity and distributional data when examined systematically [24]. Our findings extend this perspective by demonstrating that such bycatch can also retain sufficient morphological integrity and DNA quality (often preserved by cold-chain handling during arbovirus surveillance) for plant pathogen screening and phylogenetic characterization. This underscores the value of interoperable surveillance infrastructures, where standardized trapping, specimen preservation, and museum curation enable the reuse of samples beyond their original purpose. Other examples where such interoperability is relevant include detection of vectors of veterinary concern, such as Ceratopogonid biting midges or Simuliid black flies, which are frequently found in the bycatches of mosquito collections (Mackay et al., unpublished data). Within a One Health framework, the integration of biodiversity surveys, zoonotic entomology surveillance, and museum collections offers an efficient and scalable approach to detect pathogens across ecological compartments, while maximizing the scientific return of existing monitoring efforts.

Although none of the insect species in which phytoplasma DNA was detected can currently be considered competent vectors, as transmission trials were not conducted, we adopted a strict and standardized workflow to ensure reliable detection of phytoplasmas in insect bodies to document the presence of phytoplasma strains circulating in natural areas in Illinois. While these species may not represent true vectors under natural conditions and may have acquired phytoplasmas incidentally through feeding or probing activities, they can be regarded as biological accumulators that reveal the presence of phytoplasma strains in natural habitats, potentially associated with still unidentified host plants. In phytoplasma research, it is not uncommon for insect species to remain classified as ‘potential’ vectors due to the logistical challenges associated with establishing laboratory colonies and conducting controlled transmission experiments. Indeed, Weintraub et al. [41] reported approximately 200 known and potential phytoplasma vectors, and transmission competence has been experimentally confirmed for about half of them. Furthermore, other authors provided evidence that vector competence in phytoplasmas may be phylogenetically constrained [42], opening avenues for inference based on evolutionary relationships when experimental trials are not feasible. Nevertheless, all insect-based phytoplasma detections reported here should be interpreted with caution and viewed as a foundation for future experimental validation, while they still represent a valuable surveillance tool for uncovering cryptic pathogen circulation and guiding future studies aimed at identifying true vectors and reservoir hosts.

In particular, the close phylogenetic relationship between the phytoplasma detected in *Osbornellus clarus* and phytoplasmas previously reported from insect hosts associated with ‘*Ca.* P. asteris’ is consistent with the broad host range and epidemiological versatility that characterize this phytoplasma lineage worldwide [43,44,45]. *Osbornellus clarus* is considered a polyphagous species and is associated with more than one family of herbaceous host plants, including Asteraceae [46]. This species is not known as an agricultural pest or as a vector of plant pathogens. However, other species within the genus *Osbornellus* are known or suspected phytoplasma vectors. These include the Palearctic *Osbornellus horvathi*, a competent vector of Aster Yellows phytoplasma in Sicily (Italy) [47]; the Nearctic *Osbornellus borealis*, a competent vector of X-disease phytoplasma (16SrIII phytoplasma group) affecting peach in California [48]; *Osbornellus dabeki*, a potential vector of coconut lethal yellowing phytoplasma (16SrIV phytoplasma group) in Jamaica [49]; and the Nearctic *Osbornellus auronitens*, a potential vector of an Alder yellows phytoplasma-related strain (16SrV-C phytoplasma) in Switzerland [50]. In our study, the specimen of *O. clarus* was found to infect ‘*Ca.* P. asteris’, collected in a relatively closed canopy woody area with moderate-dense understory vegetation in the Sangchris Lake State Park in Central Illinois.

Although some species of *Macrosteles* are well known as competent vectors of Aster Yellows phytoplasmas (group 16SrI), other species within this genus, including *M. cristata* and *M. laevis*, have been reported as competent vectors of phytoplasmas belonging to subgroup 16SrIII-B (X-disease phytoplasma) in Europe [51]. In the Czech Republic, X-disease phytoplasma has also been detected in the native North American perennial *Echinacea purpurea* (Asteraceae) cultivated as an ornamental plant [52]. In this context, the association between *M. lepidus* and a ‘*Ca.* P. pruni’-related strain (a variant of the 16SrIII-J) detected in restored tallgrass prairie habitats in Illinois appears biologically plausible. Together, these findings suggest that species of *Macrosteles* may play a broader role in the ecology and transmission of 16SrIII phytoplasmas across both managed and natural ecosystems. Thus, further investigation into their vector competence and host plant associations may help to better understand the risk factor that this association poses. *Stictocephala bisonia* was found to be infected with ‘*Ca.* P. mali’ in apple crops in Italy [53] and carrots in Serbia [54], and was also reported to have been infected accidentally with stolbur phytoplasma in a vineyard [55] in Italy. Unfortunately, in both cases, the phytoplasma load was too low to be able to further characterize the strains. The independent detection of two distinct ‘*Ca.* P. pruni’-related strains in *M. lepidus* and *S. brevis* at two close sites in eastern Illinois suggest independent biological associations within the same landscape, given that the species belong to two different families, Cicadellidae and Membracidae, and are associated with different plant hosts. Similar sympatric occurrence of divergent ‘*Ca.* P. pruni’-related strains have been reported in both plant and insect hosts in the western USA and are thought to reflect complex transmission networks involving multiple reservoir plants and vector species [56,57,58]. These findings support the idea, consistent with previous evidence, that phytoplasma diversity in natural ecosystems is underestimated, particularly when sampling is focused primarily on symptomatic crops rather than insect communities [59].

The detection of ‘*Ca.* Phytoplasma sacchari’-related strains in *L. missellus* and *P. punctiscriptus* from a natural prairie habitat in Illinois are consistent with previous reports of 16SrXI phytoplasmas from non-agricultural systems [34]. Notably, the closest match to the *P. punctiscriptus* phytoplasma was a strain detected in the wild prairie grass *Bothriochloa laguroides* in Texas, which was also collected in a natural grassland ecosystem rather than from cultivated crops. For these prairie-associated records, neither the definitive plant reservoirs nor the competent insect vectors have been identified, and their epidemiological cycles remain under investigation. The recurrence of closely related 16SrXI-H phytoplasmas in geographically distant but ecologically similar natural habitats suggests the existence of a cryptic epidemiology involving native plants and insect communities that are largely overlooked by crop-focused surveys.

Although vector competence cannot be inferred from detection alone, the presence of 16SrXI phytoplasmas in leafhopper species collected from natural areas supports the use of insects as sentinels to reveal phytoplasma diversity in unmanaged ecosystems. The divergent and incompletely characterized strain detected in *L. missellus* further highlights that additional phytoplasma diversity remains to be discovered and will require further investigation through expanded screening and sampling efforts.

Some of the specimens analyzed here were obtained as the bycatch from CDC light traps and related suction-based surveillance devices, which are primarily optimized for monitoring host-seeking adult mosquitoes [60] rather than plant-associated Hemiptera. These traps typically use incandescent or long-wave ultraviolet (UVA) light sources, often supplemented with carbon dioxide, which attract a broad range of insects [61], including a wide taxonomic range of non-target species [62,63], but may underrepresent taxa more efficiently sampled through vegetation-based methods, sweep netting, or Malaise traps. In addition, the use of relatively coarse catch container mesh (30-mesh size; ~0.6 mm opening) in CDC light traps used during the 2022 and 2024 sampling seasons may have resulted in the loss of some very small-bodied hemipterans, particularly members of the subfamily Typhlocybinae, which are among the smallest phytoplasma-associated leafhoppers. Larger-bodied hemipteran insects that include most confirmed and putative phytoplasma vectors are unlikely to have been affected by this mesh size. In contrast, UV and BG traps deployed in this study employed finer mesh catch containers, improving retention of small-bodied taxa.

Despite these limitations, this approach offers important advantages. Leveraging existing biodiversity and mosquito surveillance infrastructure and systematically screening non-target bycatches enables cost-effective, large-scale detection of phytoplasmas across diverse natural landscapes without the need for dedicated phytoplasma-focused sampling. Combining independent monitoring efforts may mitigate bias by broadening spatial coverage and capturing a more representative snapshot of insect communities and associated pathogens across preserved natural areas. Although targeted pathogen surveillance may increase detection probability, our integrative approach strengthens ecological inference by avoiding narrow, host- or symptom-driven sampling strategies that can introduce bias. This strategy is particularly valuable for detecting pathogen circulation in natural and semi-natural habitats where host plants and vectors remain poorly characterized.

From a One Health perspective, integrating plant pathogen screening into entomological surveillance frameworks traditionally focused on human and animal health or the conservation of biodiversity creates opportunities for early detection, hypothesis generation, and cross-sectoral risk assessment. While targeted vegetation sampling and vector competence studies remain essential for resolving epidemiological cycles, opportunistic analysis of surveillance bycatch provides a powerful complementary tool for understanding pathogen ecology at the interface of plant, insect, and environmental health. Future studies will be needed to evaluate the extent to which phytoplasmas detected in natural habitats may contribute to spillover into adjacent agroecosystems in Illinois.

## Figures and Tables

**Table 1:** List of specimens collected from four sites in 2022 and 2025 during two independent surveys conducted in protected natural areas in Illinois. Phy. = phytoplasma; Coll. Code = collection code.

Species	Coordinates	Date	INHS Insect Coll. Code	Associated Phy.	qPCR Ct Value	NCBI Accession
*Macrosteles* *lepidus* ^1^	87°50′11″ W 40°05′27″ N	12 June 2025	1086365	16SrIII	21.97	PX789936
*Osbornellus clarus* ^2^	89°28′08.9″ W 39°39′26.9″ N	2 September 2022	1083960	16SrI-B	24.02	PX789937
*Latalus* *missellus* ^1^	89°51′12.5″ W 40°09′09.6″ N	20 August 2025	1083966	16SrXI-H	25.85	PX789938
*Pendarus* *punctiscriptus* ^1^	89°51′12.5″ W 40°09′09.6″ N	20 August 2025	1083969	16SrXI-H	23.21	PX789939
*Stictocephala brevis* ^1^	87°33′50.9″ W 40°03′35.6″ N	29 July 2025	1089637	16SrIII	19.08	PX789940

^1^ INHS Auchenorrhyncha biodiversity survey; ^2^ INHS mosquitoes state monitoring.

## Data Availability

The sequences generated in this study have been deposited in the National Center for Biotechnology Information (NCBI) database under accession numbers PX789936- PX789940.

## Supplementary Materials

The following supporting information can be downloaded at https://www.mdpi.com/article/10.3390/insects17040383/s1. Table S1: List of all Auchenorrhyncha in-sects tested for phytoplasma presence [13,64]; Table S2: Phytoplasma reference strains used in this paper [34,65,66,67,68,69,70,71,72,73,74,75,76,77]; Table S3: List of 16 Auchenorrhyncha insects that tested positive for phytoplasmas and gel electrophoresis signals after nested PCRs.
